# Hypolipidemic effects of lactic acid bacteria fermented cereal in rats

**DOI:** 10.1186/1476-511X-11-170

**Published:** 2012-12-11

**Authors:** Immaculata Oyeyemi Banjoko, Muinat Moronke Adeyanju, Oladipo Ademuyiwa, Olugbenga Obajimi Adebawo, Rahman Abiodun Olalere, Martin Oluseye Kolawole, Ibrahim Akorede Adegbola, Tope Adebusola Adesanmi, Tosin Oluyinka Oladunjoye, Adeyemi Adeola Ogunnowo, Ahmeed Adekola Shorinola, Oluwasetemi Daropale, Esther Bunmi Babatope, Adeboye Olufemi Osibogun, Deborah Tolulope Ogunfowokan, Temitope Adeola Jentegbe, Tinuola Gbemi Apelehin, Oluwaseyi Ogunnowo, Oluwanifemi Olokodana, Falilat Yetunde Fetuga, Morenike Omitola, Linda Adugo Okafor, Catherine Lohi Ebohon, James Oluwafemi Ita, Kazeem Ayoola Disu, Omokaro Ogherebe, Stella Uche Eriobu, Anthony Alaba Bakare

**Affiliations:** 1 Department of Biochemistry, Olabisi Onabanjo University, Ikenne-Remo, Nigeria; 2 Department of Biochemistry, University of Agriculture, Abeokuta, Nigeria; 3 Department of Biochemistry, Ben Carson School of Medicine, Babcock University, Ilishan-Remo, Nigeria

**Keywords:** Dyslipidemia, Lactic acid bacteria, Probiotics, Cereals, Fermentation

## Abstract

**Background:**

The objectives of the present study were to investigate the efficacy of the mixed culture of *Lactobacillus acidophilus* (DSM 20242), *Bifidobacterium bifidum* (DSM 20082) *and Lactobacillus helveticus* (CK60) in the fermentation of maize and the evaluation of the effect of the fermented meal on the lipid profile of rats.

**Methods:**

Rats were randomly assigned to 3 groups and each group placed on a Diet A (high fat diet into which a maize meal fermented with a mixed culture of *Lb acidophilus* (DSM 20242)*, B bifidum* (DSM 20082) and *Lb helveticus* (CK 60) was incorporated), B (unfermented high fat diet) or C (commercial rat chow) respectively after the first group of 7 rats randomly selected were sacrificed to obtain the baseline data. Thereafter 7 rats each from the experimental and control groups were sacrificed weekly for 4 weeks and the plasma, erythrocytes, lipoproteins and organs of the rats were assessed for cholesterol, triglyceride and phospholipids.

**Results:**

Our results revealed that the mixed culture of *Lb acidophilus* (DSM 20242)*, B bifidum* (DSM 20082) and *Lb helveticus* (CK 60) were able to grow and ferment maize meal into ‘ogi’ of acceptable flavour. In addition to plasma and hepatic hypercholesterolemia and hypertriglyceridemia, phospholipidosis in plasma, as well as cholesterogenesis, triglyceride constipation and phospholipidosis in extra-hepatic tissues characterized the consumption of unfermented hyperlipidemic diets. However, feeding the animals with the fermented maize diet reversed the dyslipidemia.

**Conclusion:**

The findings of this study indicate that consumption of mixed culture lactic acid bacteria (*Lb acidophilus* (DSM 20242)*, Bifidobacterium bifidum* (DSM 20082) *and Lb helveticus* (CK 60) fermented food results in the inhibition of fat absorption. It also inhibits the activity of HMG CoA reductase. This inhibition may be by feedback inhibition or repression of the transcription of the gene encoding the enzyme via activation of the sterol regulatory element binding protein (SREBP) transcription factor. It is also possible that consumption of fermented food enhances conversion of cholesterol to bile acids by activating cholesterol-7α-hydroxylase.

## Background

The applications of probiotics have been well established throughout generations. The interest in the microorganisms in the recent years emanated from the discovery of their salubrious effect in lowering plasma cholesterol. The reported health promoting ability of LAB in humans and livestock include the inhibition of pathogenic microorganisms [1,2], increase in immune response [3,4], reduction of serum cholesterol levels [5] and neutralization of food mutagens produced in the colon and halting of intestinal dysfunction [6].

Epidemiological data and clinical studies indicate a positive correlation between elevated total serum cholesterol levels, mainly reflecting the LDL-cholesterol, and the development of atherosclerosis and related cardiovascular disease [7,8]. Dietary interventions that lower the intake of saturated fat and cholesterol are established as a first line therapy to reduce LDL-cholesterol. Consumption of fermented products containing LAB has been reported to reduce cholesterol [9-12].

A combination of different mechanisms has been proposed for the cholesterol lowering ability of LAB in humans. These include: (i) interaction of bile salt hydrolase of the lactic acid bacteria with the host’s bile salt metabolism. The resultant effect is the reduction in reabsorption of bile salts, loss of feedback inhibition of the bile salt synthesis and increased conversion of cholesterol to bile salts [13-15] (ii) lowering of 3-hydroxy-3-methyl glutaryl-CoA (HMG-CoA) reductase activity, a major regulatory enzyme in cholesterol biosynthesis[6,16]. However, for the LAB to be effective in this function, they must be tolerant to the acid and bile as well as inhibit the putrefactive bacteria in the gastrointestinal tract (GIT) of the host [6].

Although the hypocholesterolemic effects of probiotics are supported by a large body of research, most of these studies were confined to lipid dynamics of the plasma/serum [17-19] with only a few studies on tissue lipids [20,21]. Cholesterol and triglycerides have been the components of major interest with very little attention being given to plasma phospholipids, erythrocyte lipid profile, as well as lipid profiles of the tissues.

Consumption of the LAB fermented dairy products serve as convenient vehicle for introducing LAB with the desired characteristics into the gut. An alternative is the consumption of fermented cereal products widely utilized in developing countries where milk is either unavailable or unaffordable. Many African foods are fermented before consumption. In general, there are two major substrates for fermentation. These are cereal grains including maize, sorghum, and millet which are fermented into alcoholic beverages such as ‘burukutu’ and ‘pito’ or non-alcoholic beverage and meals such as ‘kunnu’ and ‘ogi’ or ‘agidi’ which are taken as main course meals. The second major substrate is cassava which is also subjected to acidic fermentation to produce meals such as ‘gari’, ‘fufu’ and ‘lafun’. In almost all the cases, the fermentation is initiated by chance inoculation. Lactic acid bacteria (LAB) have been identified as the predominant microorganisms in these acid fermentations [22].

The objectives of the present study were to investigate the efficacy of the mixed culture of *Lb acidophilus* (DSM 20042), *Bifidobacterium bifidum* (DSM 20082) and *Lb helveticus* (CK 60) which are gut inhabiting microorganisms in the fermentation of maize as a model of cereal fermentation in sub-Saharan Africa and the evaluation of the effect of the fermented meal on the lipid profile of the plasma, erythrocytes, the lipoproteins as well as six selected organs of wistar strain albino rats.

## Materials and Methods

### Preparation of inoculum

Mixed culture lactic acid bacteria comprising of *Lb acidophilus* (DSM 20242)*, Lb helveticus* (CK 60) *and Bifidobacterium bifidum* (DSM20082) was cultivated on MRS agar slant (37°C, 48 hr). To harvest the grown culture, sterile distilled water (10 ml) was added and shaken vigorously to dislodge the cells into the distilled water and the cells suspension was used to inoculate the sterile maize mash.

### Preparation of mixed culture LAB fermented maize mash

Cleaned maize grains (2 kg) were ground in a disc attrition mill (3 times). The maize meal obtained was weighed (500 g) into autoclavable bags (Fisher Scientific Company, U.S.A.) and sterilized (121°C, 15 min). After cooling to about 25°C, sterile distilled water (800 ml) was aseptically added and mixed thoroughly. The packs of sterile marsh were divided into two and half of the packs were inoculated with 10 ml suspension of the mixed culture LAB while 10ml distilled water was added to the remaining half to serve as control. All the packs were incubated (37^o^, 48 hr). At the end of fermentation, the products obtained were dried in an air oven (45°C, 48 hr).

### Analysis of the fermenting marsh

To monitor the fermentation process, chemical as well as microbiological analyses were carried out at regular intervals.

### Microbiological analyses

At regular intervals (12 hrs), samples of the fermenting mash (1 g) was aseptically removed and mixed with sterile water (10 ml). The suspension obtained was serially diluted and plated in triplicate. Dilutions (10^-4^ and 10^-6^) were plated on MRS agar for total count. The MRS plates were incubated at 37°C for 48 hr. At the end of incubation, the colonies on each plate were counted and the mean of triplicate determination estimated.

### Chemical analyses

Sample of the mash (5g) was aseptically removed at regular interval and mixed with distilled water (25ml). The pH of the resulting suspension was then measured after the pH meter had been calibrated using pH 7.0 and pH 4.0 buffers. The mash suspension was then used in titration with 0.1N NaOH solution to pH 8.3. The relative amount of lactic acid present was calculated as percent lactic acid on dry matter basis [23].

### Feeding trial

Experimental protocols were conducted in accordance with guidelines of the Institutional Animal Care and Use Committee and were approved by the Animal Ethical Committee of the Department of Biochemistry, Faculty of Basic Medical Sciences, Olabisi Onabanjo University, Ikenne-Remo, Nigeria. A total of ninety-one wistar strain albino male rats obtained from the animal house of the Faculty of Basic Medical Sciences of Olabisi Onabanjo University, Ikenne-Remo, Nigeria were housed individually in cage and maintained on a 12 hr light–dark cycle. Temperature and humidity were controlled at 25°C and 60% respectively. The rats were randomly assigned to 3 groups and each group placed on a Diet A, B or C respectively after the first group of 7 rats randomly selected were sacrificed to obtain the baseline data. Thereafter 7 rats each from the experimental and control groups were sacrificed weekly for 4 weeks and the plasma, erythrocytes, lipoproteins and organs of the rats were assessed for cholesterol, triglyceride and phospholipids. The high fat diets A and B were compounded as shown in Tables 1 and 2. The maize meal incorporated into each diet was either fermented with mixed culture of *Lb acidophilus* (DSM 20242)*, Lb helveticus* (CK 60*) and Bifidobacterum bifidum (*DSM 20082) to produce high fat-fermented diet i.e. Diet A or unfermented to produce high fat-unfermented diet i.e. Diet B respectively. Diet C was commercial rat chow purchased from Ladokun Feeds, Ibadan, Nigeria. 

**Table 1:** Composition of the high fat diets

**Ingredient g/kg**	**diet**
Maize (fermented or unfermented)*	500
Full cream powdered milk	300
Ground nut cake	200
Palm oil	50
Vitamin mix	20
Mineral mix	20
Methionine	3.0
Cholesterol	10

*The maize meal incorporated into the diet was either fermented with mixed culture of *Lb acidophilus, Lb helveticus and Bifidobacterum bifidum* to produce high fat-fermented diet i.e. Diet A or unfermented to produce high fat-unfermented diet i.e. Diet B respectively.

**Table 2:** Proximate composition of the high fat diet and the commercial rat chow

**Component (%)**	High fat diet*	**Rat chow**
Carbohydrate	51.6	68.4
Protein	19.4	21.0
Fat	16.1	3.5
Vitamins	2.72	2.0
Minerals	2.72	2.0
Methionine	0.28	_
Cholesterol	0.91	_
Fibre	5.5	6.0

*Two types of high fat diet were prepared, one with mixed culture of *Lb acidophilus, Lb helveticus* and *Bifidobacterum bifidum* fermented maize meal (Diet A) and the other unfermented maize meal (Diet B).

### Biochemical Analysis

#### Plasma and lipoprotein lipid profiles

Plasma concentrations of total cholesterol and triglycerides were determined with commercial kits (Spin React S.A., Santa Colona, Sant Esteve De Bas, Spain). High density lipoprotein (HDL) cholesterol and triglycerides were determined in plasma with same commercial kits for total cholesterol and triglycerides after very low density lipoproteins (VLDL) and Low density lipoprotein (LDL) were precipitated with heparin-MnCl_2_ solution as described by Gidez et al. [24]. Total phospholipids in plasma were extracted with chloroform-methanol mixture (2:1, v/v) as described by Folch et al. [25]. Phospholipids content was then determined as described by Stewart [26].

### Organ and erythrocyte lipid profiles

Lipids were extracted from organs and erythrocytes as described by Folch et al. [25]. After washing with 0.05M KCl solution, aliquots of the chloroform-methanol extract were then used for the determination of cholesterol, triglycerides and phospholipids concentrations. An aliquot of the chloroform-methanol extract was evaporated to dryness at 60°C. 20 μl of Triton X-100/chloroform mixture (1:1, v/v) was added to dissolve the lipids and again the solvent was evaporated. Then 1ml of commercially available cholesterol kit reagent (Spin React S.A., Santa Colona, Sant Esteve De Bas, Spain) was added and vortexed. After incubation in the dark at room temperature for 30 min, cholesterol content was determined by colorimetry while determination of phospholipids followed the same procedure as described for plasma.

Triglyceride concentrations in aliquots of the chloroform-methanol extracts of the tissues were determined following the procedure described by Kriketos et al. [27].

### Determination of HMG-CoA reductase activity in the liver

HMG-CoA reductase was determined according to the method of Rao and Ramakrishnan [28] by measuring the ratio of the concentrations of 3-hydroxy-3-methyl-glutaryl CoA (HMG CoA) and mevalonate in the liver. The ratio was taken as an index of the activity of HMG CoA reductase. An increase in this ratio indicates inhibition of cholesterogenesis while a decrease suggests enhanced cholesterogenesis.

### Statistical evaluation

Results are expressed as mean ± SD. One way analysis of variance (ANOVA) followed by Tukey’s test was used to analyze the results with p < 0.05 considered significant.

## Results

### Fermentation of maize mash

The total plate count of the LAB during the fermentation of the maize mash showed that LAB population increased from 4.8 × 10^5^ CFU/g at 12 hrs after inoculation to 3.6 × 10^9^ CFU/g at the end of 48 hrs after inoculation. There was no growth in the uninoculated maize mash within the first 24 hrs. A total of 3.1 × 10^3^ CFU/g was recorded after 48 hrs (Table 3). 

**Table 3:** Total plate count (CFU/g) during fermentation of maize mash

**Time (hrs)**	**LAB inoculated maize**	**Uninoculated mash maize mash**
12	4.8 × 10^5^	0
24	2.9 × 10^9^	0
48	3.6 × 10^9^	3.1 × 10^3^

There were no significant changes in pH and total acidity of the LAB inoculated and uninoculated maize mash within the first 12 hrs (Table 4). However, at the end of the 48 hrs fermentation, the pH decreased to 4.1 ± 0.3 and the acidity rose to 2.2 ± 0.1% in the maize mash inoculated with mixed culture LAB while in the unfermented, the pH decreased to 5.8 ± 0.1 and the titratable acidity rose to 0.84 ± 0.1% within the same period. The flavour and taste of the LAB fermented maize mash were reminiscent of those of yoghurt indicative of acceptable “ogi”, the traditional cereal porridge consumed as breakfast cereal in Nigeria. 

**Table 4:** Changes in pH and total acidity in mixed culture LAB fermenting maize meal and the uninoculated meal

	**pH**		**Total Acidity (%)**	
**Time**	**Inoculated meal**	**Uninoculated meal**	**Inoculated meal**	**Uninoculated meal**
12 hrs	6.4 ± 0.1	6.3 ± 0.1	0.26 ± 0.01	0.26 ± 0.02
24 hrs	5.6 ± 0.1	6.2 ± 0.05	0.96 ± 0.02	0.42 ± 0.03
48 hrs	4.1 ± 0.3	5.8 ± 0.1	2.2 ± 0.1	0.84 ± 0.1

### Biochemical analyses

#### Plasma and lipoprotein lipid profile

**Figure 1:**
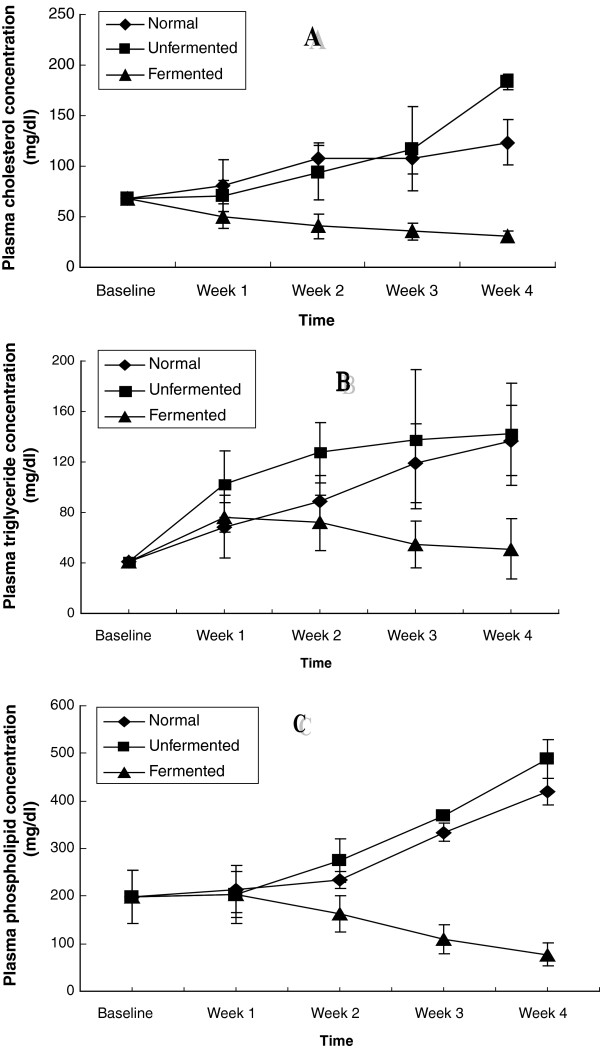
Plasma lipid concentrations of the animals – A (Cholesterol), B (Triglycerides) and C (Phospholipids).

**Figure 2:**
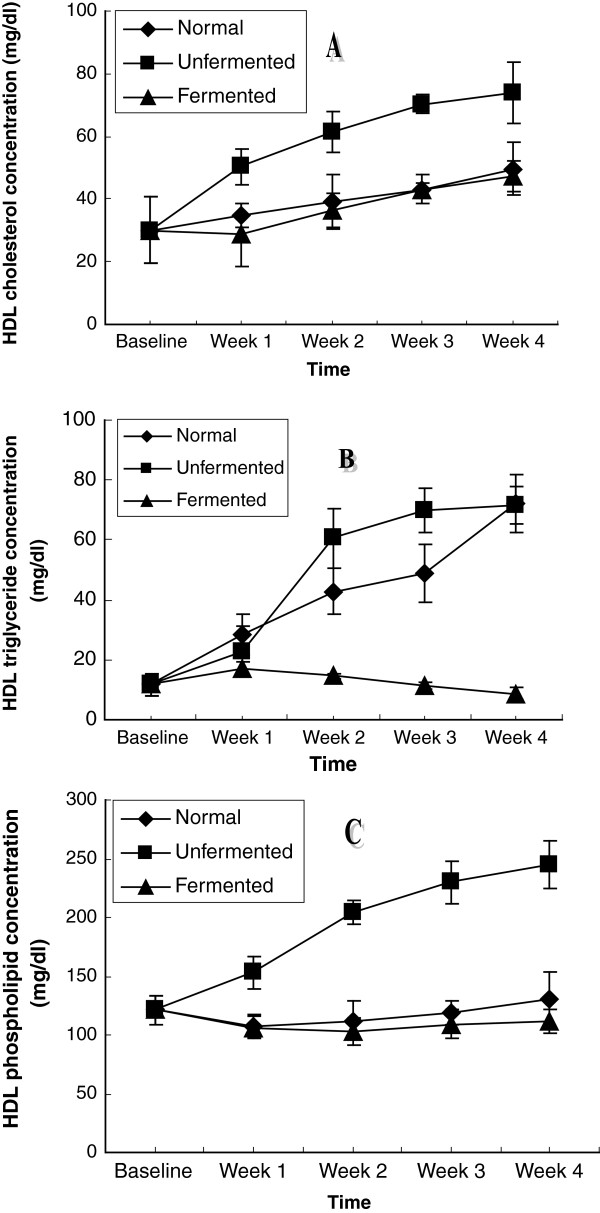
HDL lipid concentrations of the animals – A (Cholesterol), B (Triglycerides) and C (Phospholipids).

**Figure 3:**
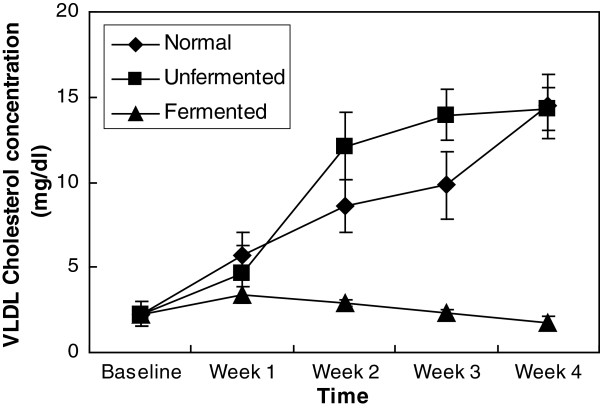
VLDL cholesterol concentration of the animals.

The results of cholesterol, triglyceride and phospholipid concentrations in the plasma and HDL as well as VLDL cholesterol are shown in Figures 1, 2, 3. Figure 1 shows the plasma lipid profiles of the animals placed on high fat diets containing LAB fermented maize mash, Diet A and unfermented maize mash, diet B as well as normal rat chow, Diet C. Consumption of the unfermented and normal diets (Diets B and C) resulted in hypercholesterolemia, hypertriglyceridemia and hyperphospholipidemia in the animals. These alterations were noticed from the first week till the end of the four-week feeding period. By the fourth week of consumption of these two diets, there was a 3-fold increase in the plasma concentrations of cholesterol, triglycerides and phospholipids. In contrast however, ingestion of the fermented diet, Diet A, reversed this trend.

The amount of lipid transported by the HDL is depicted in Figure 2. Compared to normal and fermented diets, consumption of the unfermented diet resulted in a significant increase (P < 0.05) in the amount of lipid transported by this lipoprotein during the four-week experimental period. While cholesterol increased by 2.5 fold, a 5-fold increase was observed in the triglycerides while phospholipids increased 2-fold. Although HDL cholesterol also increased as a result of the consumption of the normal and fermented diets, the increase was steady and gradual and amounted to just 68% of that of the animals consuming the unfermented diet. The amount of triglyceride and phospholipid transported by the HDL was not significantly affected by the consumption of the fermented diet.

In the VLDL (as depicted in Figure 3), consumption of the normal and unfermented diets resulted in a significant increase (P < 0.05) in the amount of cholesterol found in this lipoprotein. Comparatively, by the end of the four-week feeding period, cholesterol found in this lipoprotein was about 5 times baseline value in the animals consuming both the normal and unfermented diets, whereas consumption of the fermented diet did not seem to have any significant effect on the cholesterol content of this lipoprotein.

**Figure 4:**
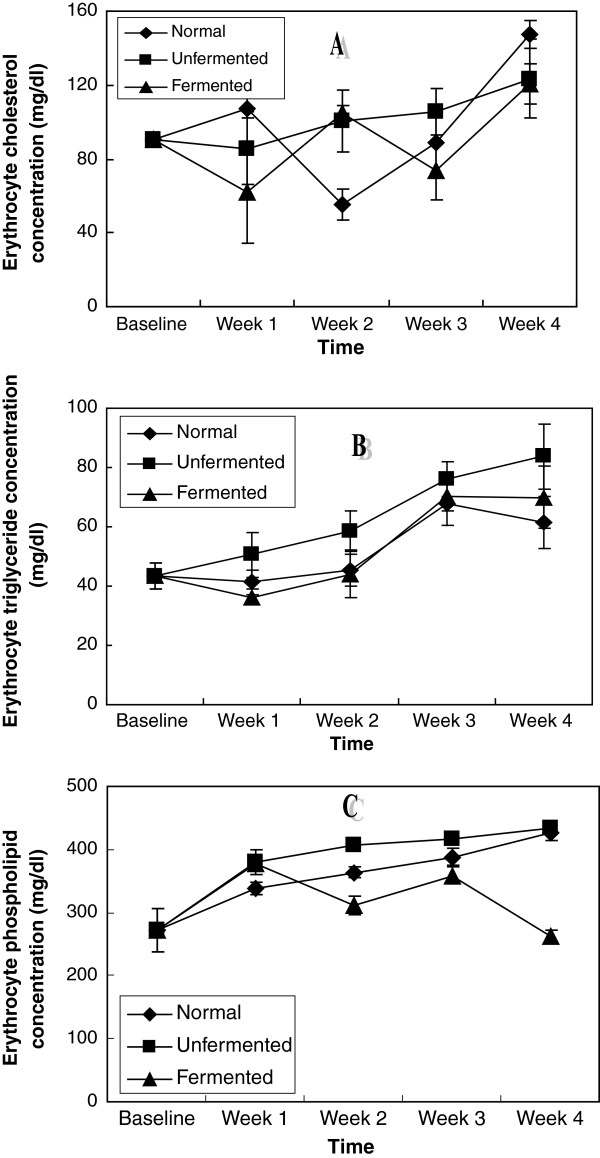
Erythrocyte lipid concentrations of the animals – A (Cholesterol), B (Triglycerides)and C (Phospholipids).

Figure 4 shows the erythrocyte lipid concentrations of the rats. While a systematic increase in all the lipids investigated characterised the effects of the consumption of the unfermented diet in the erythrocyte of these rats, the lipid lowering effects of the consumption of the fermented diet was unsystematic.

**Figure 5:**
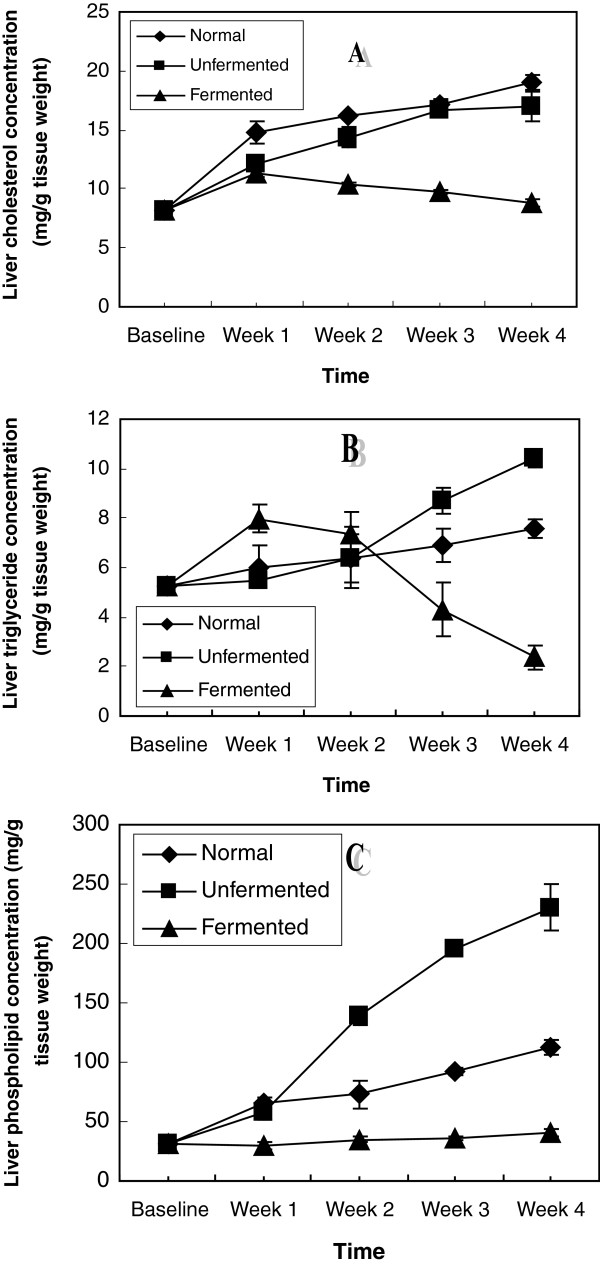
Hepatic lipid concentrations of the animals – A (Cholesterol), B (Triglycerides) and C (Phospholipids).

Hepatic lipid contents of the animals as shown in Figure 5 indicate that consumption of the normal and unfermented diets resulted in hepatic cholesterogenesis, triglyceride constipation and phospholipidosis. While the cholesterogenesis was more pronounced in rats consuming the normal diet, hepatic triglyceride constipation and phospholipidosis were more pronounced in animals consuming the unfermented diet. Consumption of the fermented diet reversed all these lipid perturbations.

**Figure 6:**
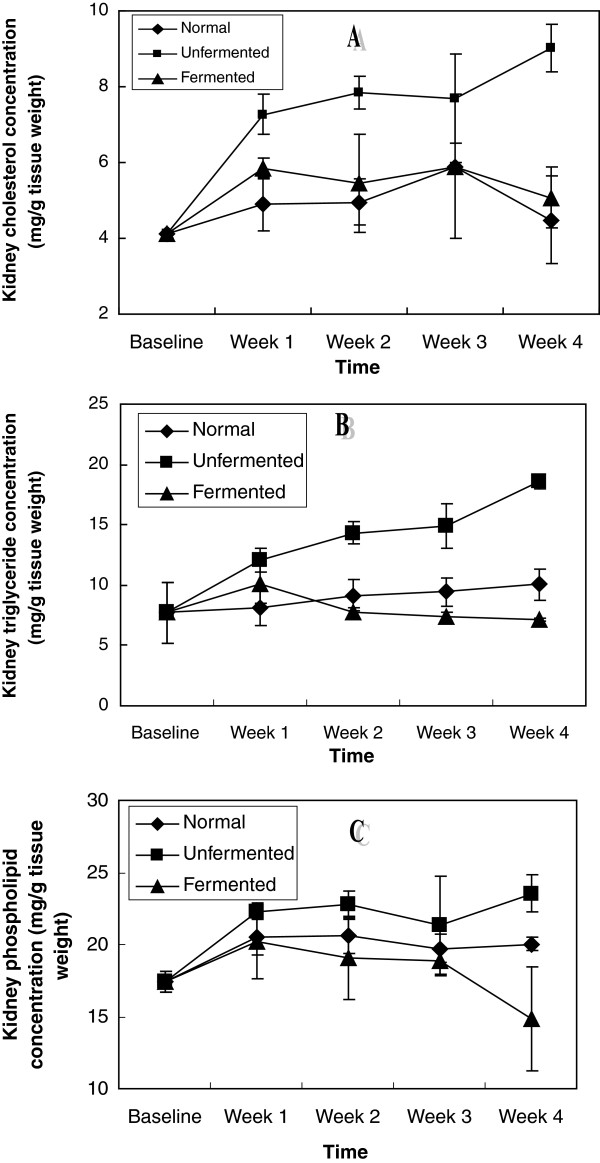
Renal lipid concentrations of the animals – A (Cholesterol), B (Triglycerides) and C (Phospholipids).

**Figure 7:**
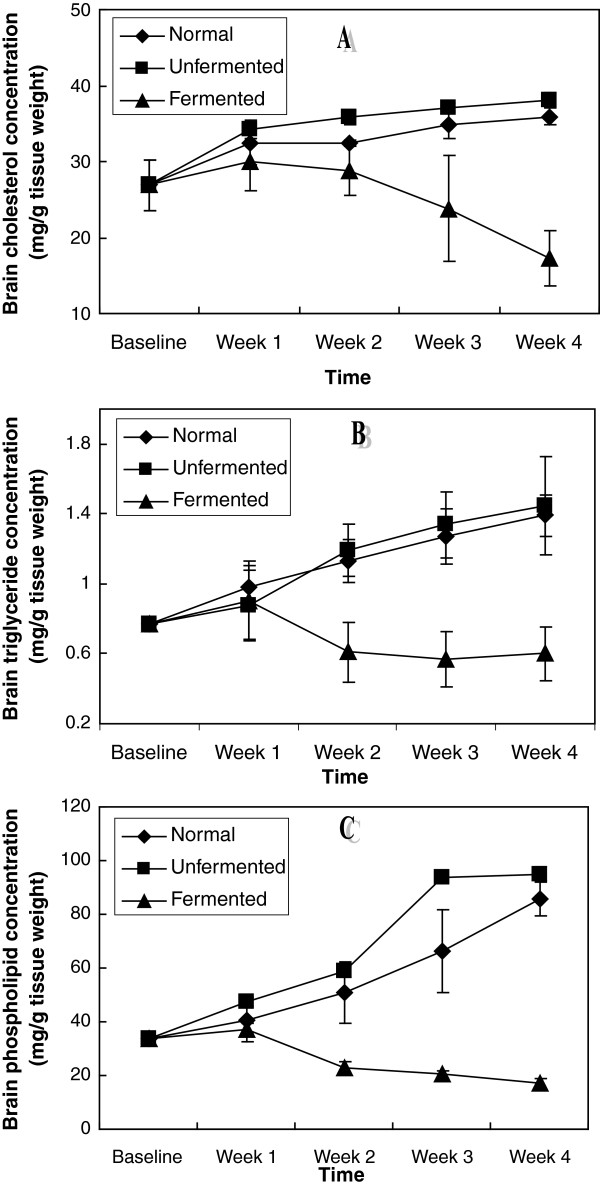
Brain lipid concentrations of the animals – A (Cholesterol), B (Triglycerides) and C (Phospholipids).

**Figure 8:**
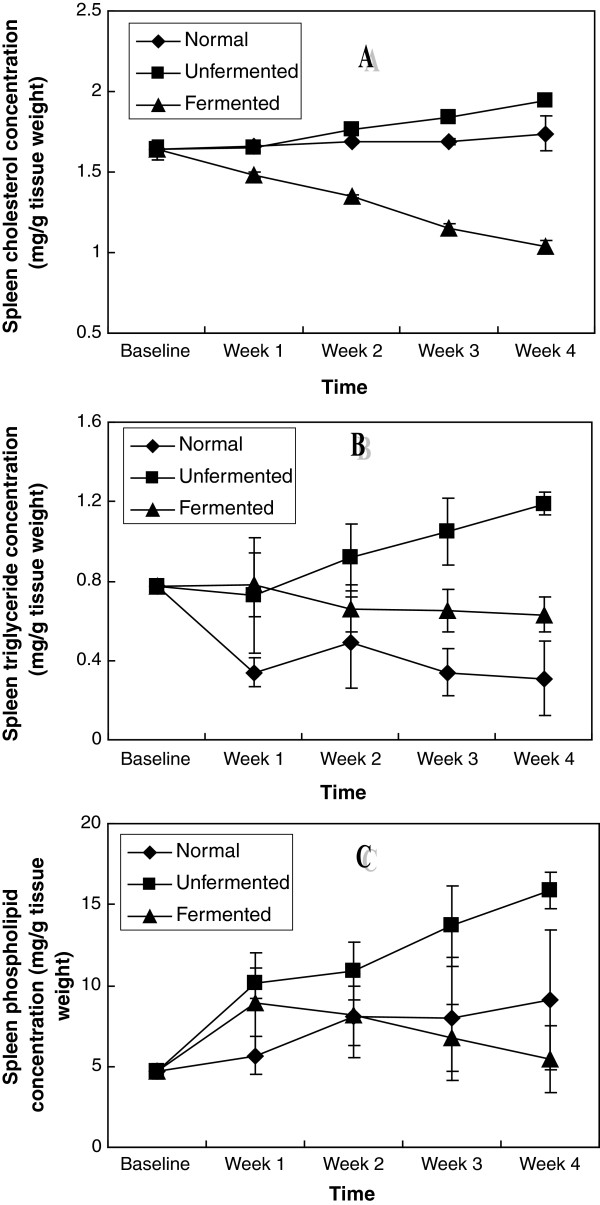
Spleen lipid concentrations of the animals – A (Cholesterol), B (Triglycerides) and C (Phospholipids).

**Figure 9:**
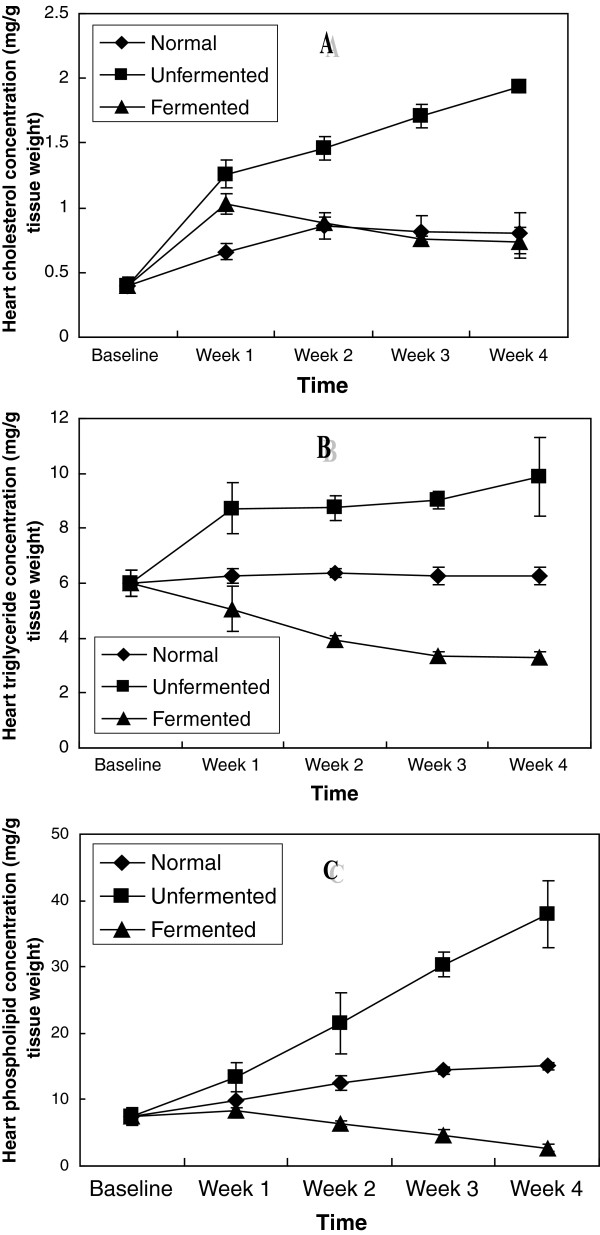
Cardiac lipid concentrations of the animals – A (Cholesterol), B (Triglycerides) and C (Phospholipids).

**Figure 10:**
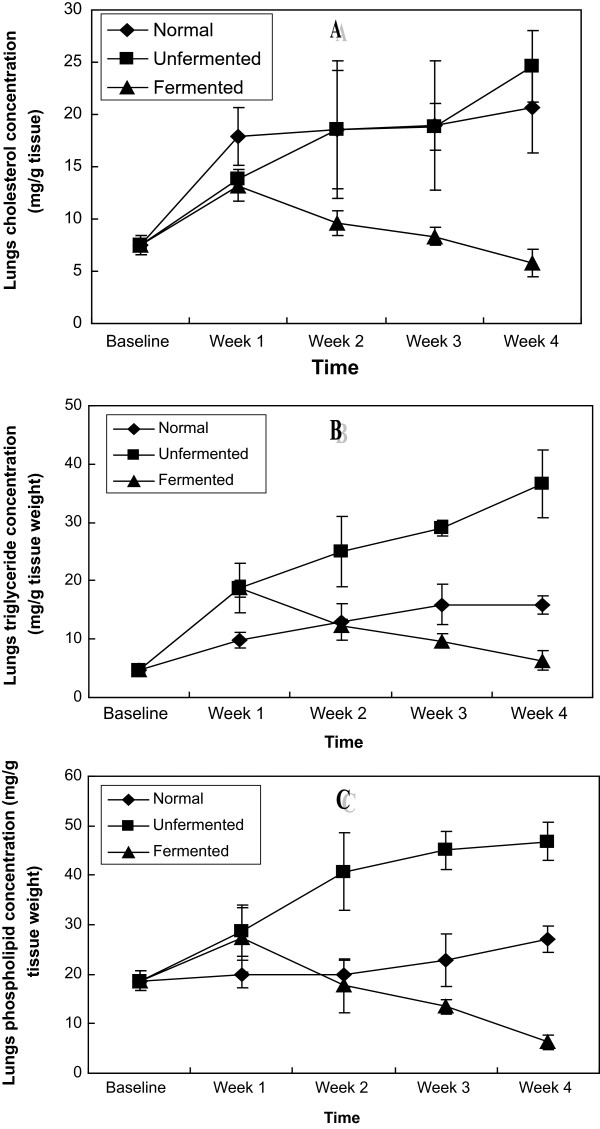
Lungs lipid concentrations of the animals – A (Cholesterol), B (Triglycerides) and C (Phospholipids).

In the extra-hepatic tissues investigated: kidney, brain, spleen, heart and lungs (Figures 6, 7, 8, 9, 10), a general pattern of lipid dynamics characterised the effects of ingestion of the diets. In these tissues, consumption of the unfermented diet resulted in excessive accumulation of cholesterol, triglyceride and phospholipids, whereas feeding the animals with the fermented diet reversed this trend.

**Figure 11:**
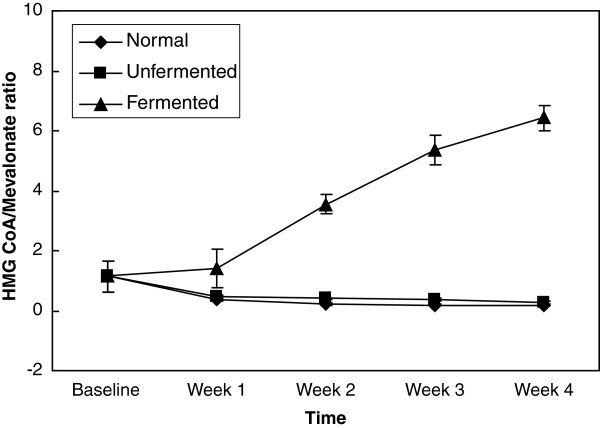
HMG CoA/Mevalonate ratio as an index of the activity of HMG CoA reductase.

Figure 11 depicts hepatic HMG CoA/Mevalonate ratio as an index of the activity of HMG CoA reductase. The activity of the enzyme increased in the liver as a result of the consumption of unfermented high fat diet, whereas the enzyme was inhibited in the rats fed with the fermented high fat diet.

## Discussion

The use of probiotics has gained scientific recognition in recent years even though their applications as functional foods have been well established throughout generations. The ability of the gut inhabiting bacteria in particular *Lb acidophilus* (DSM 20242)*, Bifidobacterium bifidum* (DSM 20082) *and Lb helveticus* (CK 60) to grow and ferment cereal into acceptable “ogi” with characteristics comparable to the traditional “ogi” in which *Lb plantarum* among other lactics is the predominant fermenting LAB [29] is a clear demonstration that the probiotics could be used in generating functional cereal foods traditionally consumed in sub Saharan Africa.

The scientific and commercial interests in these live micro-organisms derive from the range of physiological and health effects/benefits which these bacteria confer on the host, most especially on lipid metabolism [17,20]. In our study, we investigated the lipid dynamics of the plasma, erythrocytes, the lipoproteins as well as six organs in albino rats fed high fat diets, one containing mixed culture LAB fermented maize meal and the other unfermented maize meal.

The results of this study indicate that feeding rats with normal and unfermented hyperlipidemic diets resulted in perturbations in lipid metabolism in different compartments of the animals. These perturbations were characterized by up-regulation of the concentrations of the major lipids (cholesterol, triglyceride and phospholipids) not only in the plasma, but also in all the organs (liver, kidney, brain, heart, lungs and spleen) examined. These observations suggest that interaction between the normal and unfermented hyperlipidemic diets and the major lipids went beyond the level of the gastrointestinal tract each week resulting in enhanced tissue uptake and/or de novo synthesis of these lipids in all the tissues examined during the four week feeding period. While the observation of up-regulation of plasma and hepatic cholesterol and triglycerides is in agreement with the results of other workers [20], the effects of consumption of unfermented hyperlipidemic diet on plasma phospholipids on one hand, as well as the effects on the major lipids of other extra-hepatic tissues on the other hand, have not been reported. Our results thus suggest that in addition to plasma and hepatic hypercholesterolemia and hypertriglyceridemia, phospholipidosis in plasma, as well as cholesterogenesis, triglyceride constipation and phospholipidosis in extra-hepatic tissues, characterize the consumption of unfermented hyperlipidemic diets. Particularly striking was that these effects were also observed in the brain.

Mechanistically, enhanced cholesterogenesis observed in the liver and brain of the animals may be attributed to activation of HMG CoA reductase (Figure 11), while the accumulation of cholesterol by the kidney, heart, lungs and spleen during this feeding period may be mediated by an enhanced cholesterol efflux from the liver since most extra-hepatic tissues depend on the liver for their cholesterol needs [30].

The immediate substrates for triglyceride and phospholipid synthesis are the free fatty acids (FFA) [31]. They are also the major source of energy in many tissues [32]. The excessive accumulation of triglycerides in the tissues upon consumption of the normal and unfermented hyperlipidemic diets indicates that most of the absorbed FFA was directed towards the synthesis of triglycerides. This also suggests an impairment of mitochondrial β-oxidation of FFA, hence compromising energy production in these tissues.

Phospholipidosis is a lipid storage disorder in which abnormal quantities of phospholipids accumulate in various tissues [30,33]. The induction time may be a few days to several months [30,33-35]. Four major concepts have been proposed for the mechanism of induction of phospholipidosis: (1) inhibition of lysosomal phospholipase activity – this is generally regarded as the primary mechanism of induction, (2) inhibition of lysosomal enzyme transport as a result of down-regulation of genes involved in lysosomal enzyme transport, (3) enhanced phospholipid biosynthesis due to enhanced FFA availability and (4) enhanced cholesterogenesis [34]. Our data indicate that the last two mechanisms might be involved in the induction of phospholipidosis in tissues as a result of consumption of normal and unfermented hyperlipidemic diets. Although we did not determine the activity of phospholipase in this study, an inhibition of this enzyme by the diets cannot be ruled out.

Another major finding of this study was that feeding the animals with the LAB fermented maize diet reversed the dyslipidemia induced by the consumption of the unfermented hyperlipidemic diet. Several interpretations of this observation could be considered. Firstly, that the fermented diet reduced plasma cholesterol, triglycerides and phospholipids suggests that the effects of the fermented diet began at the level of the gastro-intestinal tract, inhibiting absorption of these lipids. Cholesterol reduction by lactic acid bacteria has been attributed to the ability of these bacteria to bind cholesterol in the small intestine [36]. Our data (Figure 1) indicate that in addition to cholesterol, triglycerides and phospholipids are also bound by these micro-organisms resulting in their decreased absorption and in a homeostatic response, resulting in lowering of these plasma lipids. Secondly, as regards lipid metabolism in the tissues, ingestion of the fermented diet inhibited the activity of HMG CoA reductase, thus reducing the amount of cholesterol synthesized in the liver and brain. Consistent with this was the observation of reduced cholesterol content of the extra-hepatic tissues since their cholesterol is obtained from the liver. In addition to reducing hepatic cholesterol, hepatic triglycerides and phospholipids, as well as extra-hepatic tissue lipids were also decreased by ingestion of the lactic acid fermented diet. Kitawaki et al. [20] reported that soy yogurt (prepared using lactic bacteria) fed to rats down-regulated the gene encoding Elovl 6, the elongase of fatty acid which catalyses the conversion of palmitate to stearate. Glycerol-3-phosphate acyl-transferase (involved in triacylglycerol and phospholipid synthesis), was also down-regulated [20]. Furthermore, soy yogurt ingestion up-regulated the expression of enoyl CoA isomerase, thus up-regulating β-oxidation of fatty acids [20]. The authors concluded that suppression by soy yogurt of hepatic lipids in these animals depended on the regulation of synthesis and degradation of lipids at the gene level. The same mechanisms may be assumed for the down regulation of tissue lipids observed in our study.

The fermented diet also promoted a significant increase in HDL-cholesterol. This is an important factor since HDL-cholesterol can prevent arteriosclerosis. Although the unfermented diet also promoted a significant increase in HDL-cholesterol, the increase observed in total plasma cholesterol in this group of animals apparently obviates any potential benefit that the high HDL-cholesterol might bring.

Our data on erythrocyte lipid dynamics warrant further studies. While the unfermented diet was consistent in promoting increase in erythrocyte lipids, inconsistencies observed in the effects of the fermented diet make any interpretation of data difficult.

In conclusion, the findings of this study indicate that consumption of mixed culture lactic acid bacteria (*Lb acidophilus* (DSM 20242)*, Bifidobacterium bifidum* (DSM 20082) *and Lb helveticus* (CK 60) fermented food results in the inhibition of fat absorption. It also inhibits the activity of HMG CoA reductase. This inhibition may be by feedback inhibition or repression of the transcription of the gene encoding the enzyme via activation of the sterol regulatory element binding protein (SREBP) transcription factor. It is also possible that consumption of fermented food enhances conversion of cholesterol to bile acids by activating cholesterol-7α-hydroxylase.

## Competing interests

The authors declared that they have no competing interests.

## Authors' contributions

OA and OOA conceived the study, carried out data analyses, interpreted the results and drafted the manuscript. IOB, MMA, OA, OOA and other authors conducted the study and participated in sample assays. All authors read and approved the final draft of the manuscript.
